# Post-Epidemic Chikungunya Disease on Reunion Island: Course of Rheumatic Manifestations and Associated Factors over a 15-Month Period

**DOI:** 10.1371/journal.pntd.0000389

**Published:** 2009-03-10

**Authors:** Daouda Sissoko, Denis Malvy, Khaled Ezzedine, Philippe Renault, Frederic Moscetti, Martine Ledrans, Vincent Pierre

**Affiliations:** 1 Institut de Veille Sanitaire, Cellule Interrégionale d'Epidémiologie Réunion Mayotte, Direction Régionale des Affaires Sanitaires et Sociales, Saint Denis, La Réunion, France; 2 Département des Maladies Infectieuses et Tropicales, Centre Hospitalier Universitaire de Bordeaux and Centre René Labusquière, Université de Bordeaux 2, Bordeaux, France; 3 Médecine Générale, Saint Paul, La Réunion, France; 4 Institut de Veille Sanitaire, Département International et Tropical, Saint-Maurice, France; Case Western Reserve University School of Medicine, United States of America

## Abstract

Although the acute manifestations of Chikungunya virus (CHIKV) illness are well-documented, few data exist about the long-term rheumatic outcomes of CHIKV-infected patients. We undertook between June and September 2006 a retrospective cohort study aimed at assessing the course of late rheumatic manifestations and investigating potential risk factors associated with the persistence of these rheumatic manifestations over 15 months. 147 participants (>16 yrs) with laboratory-confirmed CHIKV disease diagnosed between March 1 and June 30, 2005, were identified through a surveillance database and interviewed by telephone. At the 15-month-period evaluation after diagnosis, 84 of 147 participants (57%) self-reported rheumatic symptoms. Of these 84 patients, 53 (63%) reported permanent trouble while 31 (37%) had recurrent symptoms. Age ≥45 years (OR = 3.9, 95% CI 1.7–9.7), severe initial joint pain (OR = 4.8, 95% CI 1.9–12.1), and presence of underlying osteoarthritis comorbidity (OR = 2.9, 95% CI 1.1–7.4) were predictors of nonrecovery. Our findings suggest that long-term CHIKV rheumatic manifestations seem to be a frequent underlying post-epidemic condition. Three independent risk factors that may aid in early recognition of patients with the highest risk of presenting prolonged CHIKV illness were identified. Such findings may be particularly useful in the development of future prevention and care strategies for this emerging virus infection.

## Author Summary

Transmitted by day-biting mosquitoes, Chikungunya virus (CHIKV), causing febrile illness and joint pain, is widespread in Africa and Asia. Recent outbreaks in the Indian Ocean islands, its rapid expansion across continents, and its unusual clinical acute pattern have shed light on and brought awareness of this re-emerging virus. However, late rheumatic manifestations of this infection remain poorly examined. Thus, we have evaluated over a 15-month period the evolution and assessed potential risk factors of CHIKV-related rheumatic manifestations in a cohort of Reunion Island residents infected with CHIKV during the initial phase of the epidemic in 2005. Eligible adult patients were identified through a surveillance database. We found that rheumatic symptoms lasted for at least 15 months in 57% of the participants. Of these, 63% reported permanent trouble while 37% had recurrent symptoms. Factors such as age ≥45 years, severe initial joint pain, and presence of underlying osteoarthritis comorbidity were associated with nonrecovery over the 15-month period. Our findings suggest that long-term CHIKV rheumatic manifestations seem to be a hidden, frequent, post-epidemic condition. These findings should be considered in the development of preventive measures.

## Introduction

Chikungunya virus (CHIKV, family *Togaviridae*, genus *Alphavirus*) is a mosquito-borne virus belonging to the *Semliki Forest* serocomplex, which includes *Ross River virus (RRV)*, *O'nyong-nyong (ONN)*, *Mayaro (MAY)*, and *Barmah Forest viruses (BFV)*. Common clinical manifestations caused by these viruses include abrupt onset of fever, headache, backache, and arthralgia [1].

Since its initial identification in Newala province (Tanzania) in 1953 [2], CHIKV has been associated with numerous outbreaks, mainly in Africa [3],[4] and Asia [5],[6]. In early 2005, CHIKV was introduced into the Southwestern Indian Ocean region, probably from infected viraemic travellers arriving from Lamu (Kenya), where an outbreak started in June 2004 [7]. Subsequently, it rapidly spread across the Southwestern Indian Ocean islands (Comoros, Madagascar, Mayotte, Seychelles, Mauritius, Reunion Island), resulting in an extensive regional epidemic in 2005 and 2006 [8]–[12]. Afterward, these outbreaks' viruses expanded into Asian countries [13],[14]. Concurrently, numerous imported cases were noticed in nontropical Western countries [15],[16]. Moreover, these imported cases raised concerns about the possibility of the emergence of CHIKV infection in Europe, as *Aedes albopictus*, the mosquito vector of CHIKV in Reunion Island [17], is present in several European countries [18], including Italy, where it was first recorded in the 1990s and is particularly common [19]. Indeed, this concern was born out by documentation of an autochthonous CHIKV outbreak in the Ravenna region of Italy during the summer of 2007, which was linked to a viraemic index patient originating in Kerala, India [20].

In Reunion Island, CHIKV outbreak evolved in two-wave phenomena with a first wave between March to June 2005 and the second one from December 2005 to June 2006. The overall attack rate in Reunion Island was estimated to be 35% in mid-2006. Subsequently, CHIKV presented with an endemo-sporadic pattern until the beginning of 2007, when it disappeared. However, in spite of this, chronic rheumatic symptoms have persisted in many previously infected individuals.

Rheumatic manifestations of CHIKV infection typically consist of a febrile arthritis principally affecting the extremities (ankles, wrists, phalanges), although many others joints may be affected [21]. Patients adopt a characteristic stooped walking position which is the hallmark of the disease and from which it derives its name Chikungunya, meaning “he who walks bent over”, in the Kimakonde language of Mozambique. Although the acute rheumatic involvement is well-documented, less is known about the potential long-term clinical and functional outcome [21]–[23]. To our knowledge, the most detailed description is a cohort of 107 patients by Brighton et al. [24], which indicated that nearly 12% of patients had not fully recovered three years after initial infection. In that study, determinants of the course of rheumatic manifestations of CHIKV infections were not investigated.

In order to address these shortcomings, we have evaluated rheumatic complications in a cohort of Reunion Island residents infected with CHIKV during the initial phase of the epidemic in 2005. The study had two objectives, firstly to assess the course of rheumatic manifestations over the 15 months following the acute illness, and secondly to investigate potential risk factors associated with long-term outcome.

## Materials and Methods

### Design

This study was conducted on Reunion Island, a French territory in the Southwest Indian Ocean. We used a community-based, retrospective cohort design involving 3,539 presumptive or laboratory-confirmed cases of CHIKV illness that were reported to the local health authority during the first wave of this outbreak (March–June 2005). Recruited persons were interviewed by telephone between June 1 and September 15, 2006.

### Participants

In order to identify potentially eligible participants, we used a case notification surveillance database which has previously been described elsewhere. Patients aged more than 16 years old with serologically confirmed CHIKV infection were invited to participate. Seropositivity was defined as the presence of CHIKV-specific immunoglobulin [Ig] M antibody by IgM-capture enzyme-linked immunosorbent assay or detection of CHIKV in body fluids by amplification of CHIKV RNA using reverse transcriptase–polymerase chain reaction (RT–PCR). Other inclusion criteria included disease onset between March 1 and June 30, 2005, a telephone number accessible through the database, and provision of oral informed consent for all participants or from parents or guardians for those less than 18 years of age.

### Data Collection

Data were collected by a trained physician using a standardised structured questionnaire in French, which was developed for the purposes of the study and administered by telephone interview.

Collected information included: demographic characteristics (age, gender, educational level, employment status, marital status), major comorbidities, acute rheumatic manifestations (anatomical location, date of onset, duration of symptoms, and pain intensity), hospitalisation, treatment received (medications, physiotherapy, alternative and complementary therapies), subjective treatment satisfaction, and impact of illness on professional or household or daily activities. Moreover, chronic rheumatic manifestations (in particular, the duration of morning stiffness of ≥45 minutes as a typical sign of inflammatory pattern) at month 15 following infection were assessed. For both acute and chronic manifestations, pain intensity was evaluated using a declarative numerical rating scale (NRS) [25]. This scale could be scored between 0 and 10, with higher values indicating more severe pain. Scores were categorised into three classes (mild: 1–4; moderate: 5–6; or severe: 7–10).

### Outcomes

The primary outcome measure was self-perceived recovery from rheumatic manifestations of CHIKV infections at 15 months following disease onset. This was assessed by asking two questions with bimodal (yes/no) response modes. The first question was “Do you feel that you have made a complete recovery from joint manifestations since being diagnosed as having a CHIKV infection?” For those who replied affirmatively, a subsidiary question was asked: “Over the past eight days, did symptoms of CHIKV illness subside and subsequently recur?”. This allowed permanently asymptomatic (remitted) patients to be differentiated from those who had experienced a relapse within the past eight days. Patients replying negatively to the first question and affirmatively to the second were combined in a “persistence” group. The analysis compared this “persistence” group to a “remission” group who replied negatively to the second question.

### Statistical Analysis

To identify potential risk factors for persistence of rheumatic manifestations, the two outcome groups were compared by univariate and multivariate unconditional logistic regression. All variables were firstly assessed individually in a univariate model, and odds ratios (OR) estimated with their corresponding 95% confidence intervals (CIs) and P-values determined with the χ^2^ likelihood test. Parameters whose distribution varied between groups at a probability level of P<0.25 were retained and entered into a multivariate logistic regression analysis using a forward stepwise selection procedure. Multivariate analysis was conducted with adjustment for gender. Possible interactions and multi-colinearity were examined. If two or more potential factors risk were highly correlated, the predictor that was considered to be more clinically important was selected for entry. Finally, the goodness-of-fit of the final model was assessed using the logistic regression diagnostics procedure. A probability value of ≤.05 (two-tailed) was taken to be statistically significant. Statistical analyses were performed with STATA software, version 9 (STATA Corp., College Station, Texas, United States).

### Ethical Considerations

The survey design was approved by both the French Data Protection Authority (*Commission Nationale Informatique et Liberté*) and the National Council for Statistical Information (CNIS).

## Results

### Participants

In total, 3,539 presumptive or laboratory-confirmed CHIKV infection cases were reported between March 1 and June 30, 2005. CHIKV infection was confirmed serologically for 873 cases. Of these, 713 persons were excluded for the following reasons: no phone contact (n = 465); unknown date of CHIKV infection (n = 248). Of the remaining 160 patients who were invited to participate, 147 provided oral informed consent (92%) and constituted the study population.

This study population included 69% women and 31% men. The mean age of the patients was 52 years (SD: 15). Nearly 31% of the patients were age less than 45 years old, and 21% were more than 65 years old. Only 67 patients (45%) were working or studying at the time of the onset of their CHIKV illness. The median time elapsed between the onset of CHIKV illness and the interview was 439 days (range, 370 to 508 days). The most frequently encountered medical comorbidities were hypertension in 48 patients (33%), osteoarthritis in 38 (26%), and diabetes mellitus in 32 (22%) (Table 1).

**Table 1:** Baseline characteristics of participants with confirmed CHIKV infection (N = 147), Reunion Island, 2005–2006.

Variable	Subcategory	Result (%)
**Age in years, median (range)**		52 (16–86)
	<25	11 (7.5)
	25–44	34 (23.1)
	45–64	72 (49.0)
	≥65	30 (20.4)
**Gender**	Male	45 (31)
	Female	102 (69)
**Schooling in years**	0–6	50 (34)
	7–9	62 (42)
	≥10	35 (24)
**Employment status**	Studying	5 (3)
	Salaried employment	62 (42)
	Unemployed	17 (12)
	Retired	42 (29)
	Housewife	21 (14)
**Marital status**	Married	81 (55.5)
	Living alone (single, separated, widowed)	66 (44.5)
**Presence of comorbidities**		76 (52)
**Type of comorbidity** *	Diabetes mellitus	32 (22)
	Hypertension	48 (33)
	Osteoarthritis	38 (26)
	Chronic cardiac disease	14 (10)

*: Only comorbidities present in ≥10 percent of patients are listed.

### Disease Characteristics and Health Care Utilisation

Table 2 presents the frequency and course of rheumatic symptoms. All 147 patients reported joint pain during the initial phase of the disease. Joint pain was reported as symmetrical in 96% of patients. The principal locations of rheumatic symptoms were the ankles in 112 (76%), the wrists in 91 (62%), the knees in 65 (44%), the fingers in 79 (54%), and the toes in 84 (57%) of the patients. More than four joints were affected (polyarthritis) in 76% of the patients.

**Table 2:** Acute clinical characteristics and treatment among participants with confirmed CHIKV infection (N = 147), Reunion Island, 2005–2006.

Variable	Subcategory	Result (%)
**Time since onset in days**	Mean±standard deviation	438±38
	Median	439
	Range	370–508
**Initial rheumatic symptoms**	Joint pain	147 (100)
	Joint stiffness	134 (91)
	Join swelling	103 (70)
**Initial joint pain intensity (NRS** * **score)**	Mild	5 (3.4)
	Moderate	28 (19)
	Severe	114 (77.6)
**Main joints affected initially**	Ankles	112 (76)
	Wrists	91 (62)
	Toe joints	84 (57)
	Finger joints	79 (54)
	Knees	65 (44)
**Initial arthritis presentation**	Oligoarthritis (2–4 joints)	35 (24)
	Polyarthritis (>4 joints)	112 (76)
**Symptom duration in days**	<15	34 (23)
	15–30	20 (14)
	>30	93 (63)
**Lifestyle impact**	Missed school or work**	40 (66.6)
	Household or daily activities	113 (75)
**Medical care**	Hospitalisation	22 (15)
	NSAID***	114 (78)
	Paracetamol	137 (93)
	Corticosteroids	34 (23)
	Medicinal plants	68 (46)
	Physical or occupational therapy	29 (20)
**Perceived satisfaction with treatment**	NSAID	41/114 (36)
	Paracetamol	47/137 (34)
	Corticosteroids	26/34 (76)
	Medicinal plants	21/68 (31)
	Physical or occupational therapy	4/29 (14)

*: Numerical rating scale.

**: among employed.

***: nonsteroid anti-inflammatory drug.

Overall, 137 (93%) declared having been prescribed paracetamol. Among these, 106 (77%) reported a combination of paracetamol with a nonsteroid anti-inflammatory drug (NSAID). Nearly half of the patients (46%) were taking medicinal plants as complementary therapy. Corticosteroids were prescribed to 23% of patients at some stage of the disease course. Approximately 35% of patients reported being satisfied with their medication, with the exception of those taking corticosteroids, who were more satisfied (76%).

During the acute phase, 22 (15%) patients were hospitalised with a median length of stay of five days (range: 1 to 22 days).

### Disease Course, Impact, and Risk Factors for Persistence

Overall, 63 (43%) declared being completely recovered at the time of the interview, while 31 (21%) reported experiencing at least one episode of recurrence, and 53 (36%) stated having permanent symptoms (Table 3). Thus, the “remission” group consisted of 63 patients and the “persistence” group included 84 individuals.

**Table 3:** Rheumatic disease course over 15-month period and impact among participants with confirmed CHIKV infection (N = 147), Reunion Island, 2005–2006.

Variable	Subcategory	Result (%)
**Occurrence of symptoms**	None	63 (43)
	Fluctuating	31 (21)
	Persistent	53 (36)
**Nature of rheumatic symptoms**	Pain	84 (57)
	Morning stiffness ≥45 minutes	61 (41)
	Swelling	22 (15)
**Impairment of activities of daily living** * **in days**	<90	50 (59.5)
	90–180	24 (28.6)
	>180	10 (11.9)
**Joint pain intensity (NRS** ** **score)**	Mild	70 (83.3)
	Moderate	13 (15.5)
	Severe	1 (1.2)

*: In patients with persistent symptoms.

**: numerical rating scale.

In the “persistence” group, all subjects reported the presence of joint pain, the latter symptom being associated with morning stiffness for ≥45 minutes in 73% of that group. Pain intensity was rated as mild to moderate by the majority of patients (98.8%). In this group, a reduction of daily activities as a result of their illness was reported for >90 days in 40% of subjects.

The univariate analysis comparing the “persistence “and “remission” groups is presented in Table 4. Subjects aged ≥45 years were significantly more likely to belong to the “persistence” group than those aged <45 years (OR = 4.2, 95% CI 1.9–9.3). The presence of an underlying illness was also found to be more frequent in this group (OR = 3.0, 95% CI 1.5–5.9), in particular the presence of hypertension or osteoarthritis. On the other hand, no such association was observed for other comorbidities. Finally, the severity of pain at disease onset was strongly associated with persistence (OR = 3.6, 95% CI 1.6–8.1).

**Table 4:** Factors associated with persistence of rheumatic manifestations over 15-month period (univariate logistic regression analysis) among participants with confirmed CHIKV infection (N = 147), Reunion Island, 2005–2006.

Variable	Subcategory	Remission Group, Result (%)	Persistence Group, Result (%)	Odds Ratio (95 CI)*
**Gender**	Male	23 (37)	22 (26)	Reference
	Female	40 (63)	62 (74)	1.6 (0.8–3.3)
**Age in years**	<45	26 (41)	12 (14)	Reference
	≥45	37 (59)	72 (86)	4.2 (1.9–9.3)
**Education in years**	0–6	20 (32)	30 (36)	Reference
	7–9	29 (46)	33 (39)	0.8 (0.4–1.6)
	≥10	14 (22)	21 (25)	1.0 (0.4–2.4)
**Presence ≥1 comorbidity**	No	40 (63)	31 (37)	Reference
	Yes	23 (37)	53 (63)	3.0 (1.5–5.9)
**Hypertension**	No	49 (78)	50 (60)	Reference
	Yes	14 (22)	34 (40)	2.4 (1.1–4.9)
**Diabetes mellitus**	No	54 (86)	61 (73)	Reference
	Yes	9 (14)	23 (27)	2.3 (0.9–5.3)
**Osteoarthritis**	No	54 (86)	55 (65)	Reference
	Yes	9 (14)	29 (35)	3.2 (1.4–7.3)
**Initial pain intensity (NRS** ** **score)**	NRS<7	22 (35)	11 (13)	Reference
	NRS≥7	41 (65)	73 (87)	3,6 (1,6–8,1)

*: 95% confidence interval.

**: numerical rating scale.

In the multivariate model, three variables remained independently associated with persistence, namely age ≥45 years, initial severity of joint pain, and comorbid osteoarthritis (Table 5).

**Table 5:** Factors associated with persistence of rheumatic manifestations over 15-month period (multivariate logistic regression analysis) among participants with confirmed CHIKV infection (N = 147), Reunion Island, 2005–2006.

Variable	Total	Remission Group, Result (%)	Persistence Group, Result (%)	Odds Ratio (95%CI)*	P-Value
Age ≥45 y	109	37 (34)	72 (66)	3.9 (1.7–9.7)	0.002
Osteoarthritis	38	9 (24)	29 (76)	2.9 (1.1–7.4)	0.029
NRS**≥7	114	41 (36)	73 (64)	4.8 (1.9–12.1)	0.001

*: 95% confidence interval.

**: numerical rating scale.

## Discussion

Following the massive epidemic of CHIKV infection in the Southwest Indian Ocean region in 2005–2006, local and international awareness of this condition increased markedly [9],[16],[26]. In previous outbreaks, acute CHIKV illness was well-documented [27], but chronic post-infectious conditions related to CHIKV disease had received little attention. Our community-based study reports original data on the course of chronic rheumatic manifestations among 147 patients infected during an early phase of Reunion Island's epidemic event. In this study, only 43% of patients reported full remission 15 months after acute infection. Nearly half of the patients with persistent rheumatic pain were impaired in carrying out daily or household activities for more than three months. Furthermore, our findings indicate that chronic rheumatic manifestations of CHIKV infection were independently associated with older age at the time of infection, severe initial pain, and the presence of comorbid osteoarthritis. The identification of such risk factors could be relevant for early recognition and management of patients at risk for developing persistent rheumatic symptoms.

Few previous studies have addressed the long-term clinical outcome of CHIKV illness. In a small case series of 28 residents of Pretoria with confirmed CHIKV infection, Fourie and Morrison [22] reported that 73% of the subjects experienced severe arthralgia in the acute phase of the illness whereas 18% reported longstanding rheumatic symptoms as long as 20 months after infection. In another South African study, Brighton et al. [24] reported that 12% of 107 subjects continued to experience fluctuating or persistent rheumatic manifestations three to five years after acute CHIKV illness. Compared to the present study, the lower proportion rate of persistent rheumatic manifestations may be in part related to the interval of three to five years between illness onset and the evaluation. Indeed, the prevalence of rheumatic symptoms associated with Semliki Forest serocomplex viruses, namely Ross River virus, is known to decline over time [28]. Accordingly, it appears speculative to impute the lower prevalence principally to the population age distribution in the Brighton study. Despite this discrepancy, Brighton et al. [24] suggested that symptom persistence seemed more frequent in patients aged more than 40 years, consistent with the present findings. In addition, a recent study conducted on Reunion Island in the setting of a hospital-based recruitment had evaluated at month 18, 88 of 202 (44%) CHIKV-infected patients who fulfill the inclusion criteria [29]. Among these, 56 patients (63.6%) reported persistent arthralgia, with 29 (51.8% of the 56) ascertaining a history of arthralgia before CHIKV illness. As noted by the authors, these results are of interest for the influence of a previous history of rheumatic manifestations in the course of persistent arthralgia in patients with CHIKV infection. However, with regard to the very low rate of evaluated patients among those recruited, a meaningful selection bias had probably induced an overestimation of the proportion of persistent arthralgia. Moreover, it can be hypothesized that the patients who had totally recovered from CHIKV illness are less likely to comply and participate in such a followup study.

Our observation of symptom persistence in 57% of subjects is reminiscent of findings from longitudinal studies of rheumatic manifestations of other alphaviral infections reported from Australia (RRV) [30],[31] and Finland (*Sindbis virus*, SINV) [32],[33], although others have pointed out that other confounding factors may influence the duration of rheumatic symptoms in such diseases [34]. Hence, we recognize it can be very challenging to differentiate between osteoarthritis and Chikungunya-related arthralgia. This fact has been highlighted by evaluations conducted through clinical series of CHIKV-infected patients which clearly identified a wide range of rheumatologic manifestations including tenosynovitis and lesions involving previously injured bones or joints [35]. Nonetheless, the presence of osteoarthritis conditions before CHIKV infection appears to be an independent risk factor for developing late rheumatism manifestations. Thus, further investigations should elucidate the pathophysiologic patterns underlying this relationship.

With respect to symptom management, an important finding was that only one-third of patients reported being satisfied with the most prescribed class of treatment (i.e., NSAIDs). On the other hand, treatment satisfaction with corticosteroids at any stage of the disease was considerably higher. However, it should be emphasised that caution should be exercised when using corticosteroids in such patients (infected with a virus). Nonetheless, the study demonstrates that in practice, treatment of rheumatic symptoms of CHIKV illness was generally inadequate. However, the role of inadequate treatment as a potential risk factor for persistence could not be evaluated in the present study. This issue would be worth addressing in future studies.

This study has certain limitations that should be taken into consideration when interpreting the results. Firstly, even if the participation rate was high, only one-fifth of the eligible subjects were invited to participate since telephone contact was not available for the other subjects. Additionally, subjects under 16 years old were not eligible for the study for the reason that they are generally less likely to answer a phone-standardized questionnaire. Therefore, these findings may not be generalizable to all cases of CHIKV infection that occurred on Reunion Island. Secondly, data were collected by self-report, which precluded symptom ascertainment by objective clinical examination. Moreover, with subjective self-report, recall bias leading to underestimation of either current or past symptoms cannot be excluded. Finally, this evaluation was conducted while the second wave of the outbreak was ongoing in 2006 [9]; in this context, the constitution of a CHIKV antibody negative controls group for the setting of a case-control study design appeared irrelevant. This restriction is a break for the estimation of long-term absolute rheumatic-morbidity attributable to CHIK-illness. These limitations, however, should be tempered, as retrospective studies allow giving a rapid and prompt response to health authorities by providing consistent and relevant information and tools that will help to adapt delivered messages to the population and the medical staff in the continuing context of Reunion Island's longstanding outbreak.

The results of this study highlight the fact that, following the CHIKV outbreak in 2005 on Reunion Island, a substantial proportion of persistent and disabling residual rheumatic symptoms could be identified for at least 15 months after infection onset, especially in individuals ≥45 years of age. We also identified two other independent risk factors associated with persistence of rheumatic symptoms that may be particularly useful in the development and improvement of future prevention and care strategies for this emerging viral infection. Our findings also draw attention to the importance of assessing management issues such as strategies for supportive treatment of CHIK illness in further studies. Finally, these studies should be designed in order to estimate the magnitude of chronic rheumatic illness directly attributable to CHIKV infection and its potential effect on quality of life over a prolonged period.

## Supporting Information

Alternative Language Abstract S1 Translation of the Abstract into French by Daouda Sissoko
(0.01 MB DOC)
