# Supplementary material for: Post-Epidemic Chikungunya Disease on Reunion Island: Course of Rheumatic Manifestations and Associated Factors over a 15-Month Period
Source: PLoS Negl Trop Dis. 2009 Mar 10;3(3):e389. doi: 10.1371/journal.pntd.0000389 (PMC2647734; doi:10.1371/journal.pntd.0000389)
Supplement: Alternative Language Abstract S1 — Translation of the Abstract into French by Daouda Sissoko (0.01 MB DOC) [file pntd.0000389.s001.doc]

French Translation of the Abstract by DS
Bien que la les manifestations aiguës liées au virus du chikungunya (CHIKV) soient bien documentées peu de données relatives aux manifestations rhumatologiques persistantes existent. Nous avons mené entre juin et septembre 2006 une enquête de cohorte visant à évaluer la fréquence des manifestations articulaires persistantes après l'infection par le CHIKV et investiguer les facteurs liés à leur persistance des manifestations articulaires durant les 15 mois suivant l'infection. 147 personnes âgées de plus de 16 ans et ayant une infection CHIKV confirmée survenue entre le 1er mars et le 30 juin 2006 (identification à travers la base de surveillance de l'épidémie ont été interrogé par téléphone). Cette étude a mis en évidence une proportion élevée de manifestations articulaires au sein de cette cohorte puisque 84 participants (57%) rapportaient des manifestations articulaires 12 à 18 mois après l'infection. Parmi ces 84 patients, 53 (63 %) rapportaient des troubles permanents et 31 (37 %) des troubles occasionnels. Ajustés sur le sexe, l'âge  45 ans (OR 3,9 ; IC 95% 1,7–9,7), la sévérité des douleurs articulaires initiales (OR 4,8; IC 95% 1,9–12,1) et les antécédents d'arthrose (OR 2,9; IC 95% 1,1–7,4) étaient retrouvés significativement associés à la persistance des troubles. Ces résultats suggèrent que les manifestations rhumatologiques post CHIKV semblent être fréquentes. Trois facteurs potentiels de risque d'évolution vers la persistance ont été identifiés. Ces résultats devraient permettre de mieux cibler à l'avenir les personnes à risque d'évolution vers la chronicité et demeurent également pertinents dans le développement de futures stratégies de prévention et de prise en charge des patients atteints de cette infection.
